# Evaluation of the Biological Potential of *Himanthalia elongata* (L.) S.F.Gray and *Eisenia bicyclis* (Kjellman) Setchell Subcritical Water Extracts

**DOI:** 10.3390/foods11050746

**Published:** 2022-03-03

**Authors:** Inês Gomes, Helena Rodrigues, Carla Rodrigues, Marta Marques, Paula Paíga, Alexandre Paiva, Pedro Simões, Virgínia Cruz Fernandes, Mónica Vieira, Cristina Delerue-Matos, Cristina Soares, Clara Grosso

**Affiliations:** 1 LAQV, REQUIMTE, Instituto Superior de Engenharia do Porto, Instituto Politécnico do Porto, Rua Dr. António Bernardino de Almeida 431, 4249-015 Porto, Portugal; 10180291@ess.ipp.pt (I.G.); up201805071@edu.fc.up.pt (H.R.); 1190133@isep.ipp.pt (C.R.); pcpa@isep.ipp.pt (P.P.); virginiacruz@graq.isep.ipp.pt (V.C.F.); cmm@isep.ipp.pt (C.D.-M.); cmdss@isep.ipp.pt (C.S.); 2 Ciências Químicas e das Biomoléculas/CISA, Escola Superior de Saúde, Instituto Politécnico do Porto, Rua Dr. António Bernardino de Almeida 400, 4200-072 Porto, Portugal; mav@ess.ipp.pt; 3 LAQV, REQUIMTE, Departamento de Química, Faculdade de Ciências e Tecnologia, Universidade NOVA de Lisboa, Quinta da Torre, 2829-516 Caparica, Portugal; ma.marques@campus.fct.unl.pt (M.M.); abp08838@fct.unl.pt (A.P.); pcs@fct.unl.pt (P.S.)

**Keywords:** iodine, Maillard reaction, neuroprotection, nitrosative stress, oxidative stress, pesticides, pharmaceuticals, seaweeds, total phenolic content

## Abstract

Neuroprotection is a need that remains unmet in treating chronic neurodegenerative disorders, despite decades of extensive research. To find new neuroprotective compounds, extracts of *Himanthalia elongata* (L.) S.F.Gray and of *Eisenia bicyclis* (Kjellman) Setchell were obtained through subcritical water extraction applying a four-step temperature gradient. The fractions obtained were screened against brain enzymes involved in neurodegenerative etiology, namely in Alzheimer’s and Parkinson’s diseases, and against reactive oxygen and nitrogen species, all contributing factors to the progression of neurodegeneration. Results showed no significant enzyme inhibition but strong radical scavenging activities, particularly in the fourth fraction, extracted at the highest temperature (250 °C), highlighting their ability to retard oxidative and nitrosative stresses. At higher temperatures, fractions were composed of phenolic compounds and Maillard reaction products, a combination that contributed to their antioxidant activity and, consequently, their neuroprotective properties. All fractions were evaluated for the presence of iodine, 14 organochlorine and 7 organophosphorus pesticides, and pharmaceuticals used in Alzheimer’s and Parkinson’s diseases (14), psychiatric drugs (8), and metabolites (8). The fractions studied did not present any of the screened contaminants, and only fraction 1 of *E. bicyclis* should be used with caution due to iodine content.

## 1. Introduction

Seaweeds are a group of photosynthetic organisms found worldwide in marine ecosystems. They are taxonomically grouped into three Phyla based on their pigmentation: brown algae (Phylum Ochrophyta), red algae (Phylum Rhodophyta), and green algae (Phylum Chlorophyta). This large group of organisms is highly heterogenous and diverse, including about 11,000 species of seaweeds, of which 7500, 2000, and 1500 are red, brown, and green, respectively [1,2].

Seaweeds are employed in many maritime countries as food and fertilizer and as raw material for different purposes, e.g., pharmaceutical, cosmetic, bioenergy, and chemical industries. Consequently, their global annual production is progressively growing, amounting to 31.2 million tons (fresh weight) in 2016—mostly from Asian countries—and has more than doubled over the past 20 years. Predictions suggest that the annual production can increase by up to 20 million tons by 2050 [3].

One example of a highly valued alga in Europe is *Himanthalia elongata* (L.) S.F.Gray. *H. elongata*, also known as sea spaghetti or thongweed, which is a brown alga found in the Baltic Sea, the North Sea, and the northeast Atlantic Ocean from Scandinavia, through Ireland, and south to Portugal, and has been integrated into different foods thanks to its rich nutritional and gastronomic value [4,5]. *Eisenia bicyclis* (Kjellman) Setchell is another edible brown seaweed. It is extensively distributed in South Korea and Japan and is produced for commercial purposes, such as a carrageenan source and traditional medicine usually mixed with *Ecklonia cava* Kjellman and other seaweeds [6].

Given the growing demand for seaweeds and their extracts, scientists recognized the need to assess their safety [7,8]. Seaweeds are unavoidably exposed to the abundant presence of multiple contaminants deriving from natural and anthropogenic sources [8,9,10]. Pesticides are extensively used in agricultural and aquaculture activities to control pests and diseases [11]. Although the human and the animals’ health benefits of using pharmaceuticals are recognized, their presence as emerging contaminants in the environment is a subject of increasing concern [12]. Pharmaceuticals have been found in rivers [13], oceans [14], groundwater [15], lakes [16], drinking water [17], soils, and sediments [15,18]. Regarding legislation, the European Parliament Regulation (EC) No. 396/2005 [19] sets maximum residue levels (MRL) for some pesticides in edible seaweeds, but in the case of pharmaceuticals, no regulation has been set.

Seaweeds are rich sources of structurally diverse bioactive molecules with great pharmaceutical and biomedical potential, such as anticoagulant, antiviral, antioxidant, anti-allergic, anticancer, anti-inflammatory, anti-obesity, and neuroprotective effects [2,20].

The search for more efficient therapies to slow the rate of progression of a neurodegenerative disease is still a pressing need in the treatment of chronic diseases such as Parkinson’s disease (PD) and Alzheimer’s disease (AD), despite more than 30–40 years of extensive research [21]. The intricate and multifactorial quality of neurodegenerative disorders suggests that interventions simultaneously targeting multiple risk factors and mechanisms at an early phase of the pathologies are potentially more efficacious [21]. Current research has been highly focused on cholinesterase (ChE) enzymes, a group of esterases capable of hydrolyzing choline esters, such as acetylcholine (ACh). There are two types of ChEs, acetylcholinesterase (AChE) and butyrylcholinesterase (BuChE), and their inhibition may be one of the most realistic approaches to treat AD symptoms. Most drugs developed to treat AD, namely galantamine, rivastigmine, donepezil, and the discontinued tacrine, are ChE inhibitors [2,22]. In addition, some bioactive seaweed compounds have been shown to provide mixed type ChE (AChE and BuChE) inhibition [23].

Mitochondrial dysfunction is another manifestation associated with the pathogenesis of several ageing-related neurodegenerative diseases, particularly PD and AD. Under normal circumstances, various antioxidants in neurons counteract adverse responses; however, the dramatic increase in the production of reactive oxygen species (ROS) caused by mitochondrial dysfunction overwhelms the endogenous antioxidative mechanisms, creating oxidative stress and eventually resulting in neuronal apoptosis. Moreover, the human brain is prone to these effects due to its high oxygen consumption, particularly with highly vulnerable neurons in the substantia nigra (midbrain). Superoxide anion radical (O_2_^•−^) and hydrogen peroxide (H_2_O_2_) have been identified as critical players in a marked reduction in neuronal function and viability [24,25].

Nitric oxide (^•^NO) metabolism also contributes to oxidative and nitrosative stresses. Although ^•^NO is an important signaling molecule, it can react with other ROS to produce reactive nitrogen species (RNS). For instance, ^•^NO can react with O_2_^•−^ to form peroxynitrite (ONOO^−^) that can subsequently be converted to highly toxic intermediates such as nitrogen dioxide (NO_2_), carbonate (CO_3_^•−^), and hydroxyl (^•^OH) radicals. Moreover, ^•^NO affects cell survival through S-nitrosylation: a reversible alteration of cysteine (cys) residues in proteins to form the corresponding nitrosothiol, which regulates gene transcription, vesicular trafficking, receptor-mediated signal transduction, and apoptosis. Indeed, some neuroprotective proteins are modulated by S-nitrosylation, thus suggesting that nitrosative stress is an important contributor to the development of neurodegeneration [26,27].

Phenolic compounds are an example of essential molecules, often found in seaweeds, which confer protection against diseases involving oxidative and nitrosative stresses, namely due to their antioxidant and anti-inflammatory properties and their ability to chelate metal ions [28]. Moreover, recent studies reveal that some polyphenols may contribute to other biological effects besides their antioxidant and radical scavenging properties [28,29].

One crucial aspect to consider when working with bioactive molecules is their extraction technique. Several extraction methods are available, but the conventional ones are still the most used [30,31]. Nonetheless, these methods carry several drawbacks since they are laborious, time-consuming, and can promote the degradation of some of the desired compounds. Furthermore, they use large amounts of solvents that often produce toxic, volatile, and flammable residues, thus significantly contributing to environmental pollution and the greenhouse effect [30,31]. Consequently, green or environmentally friendly methods are being developed, presenting many advantages compared to their conventional counterparts: shorter extraction times, reduced energy consumption, fewer negative environmental impacts, and increased safety [32].

One such green process is subcritical water extraction (SWE). SWE uses liquid water at high temperatures (over 100 °C) and pressures above the corresponding vapor pressure. The water dielectric constant decreases when the temperature increases, while also lowering the polarity, viscosity, and surface tension, whereas the ionic product increases [33]. To sum up, the water behaves like organic solvents while allowing for a safer and faster extraction, better yield, and environmentally friendly conditions. All things considered, this extraction method has become increasingly popular, in part due to its unique solvation properties, which can be altered by changing the temperature [34,35,36,37,38,39].

The present work focuses on the biological properties of *H. elongata* and *E. bicyclis* fractions obtained through SWE and their neuroprotective effects and antioxidant capacity. In addition, the presence and total content of bioactive relevant biomolecules, mainly phenolic compounds and Maillard reaction products, and the presence of environmental pollutants (pesticides and pharmaceuticals) and iodine were also evaluated.

## 2. Material and Methods

### 2.1. Samples

*Himanthalia elongata* (L.) S.F.Gray and *Eisenia bicyclis* (Kjellman) Setchell were supplied dried by Algamar (Pontevedra, Spain) and Próvida (Mem Martins, Sintra, Portugal), respectively. First, after samples’ hydration for 5 min in salted water (35 g NaCl/L), they were rinsed in ultrapure water to remove NaCl. Then, they were dehydrated at 41 °C (Excalibur, model 4926T, Dublin, Ireland) for 18 h and ground to obtain particles in the 1–2 mm range.

### 2.2. Subcritical Water Extraction of Seaweeds

SWE of the seaweeds was performed using equipment described elsewhere [40]. Briefly, the extraction conditions were as follows: ca. 20 g of seaweed was placed in the reactor, and the pressure (100 bar) and water flow rate (10 mL/min) were kept constant during the experiment. After a specific time, the desired temperature in the reactor was reached, and the extract leaving the reactor was collected: first fraction—from room temperature to 90 °C (ca. 100 min), second fraction—from 90 to 140 °C (ca. 90 min), third fraction—from 140 to 190 °C (ca. 90 min), and fourth fraction—from 190 to 250 °C (ca. 100 min).

All fractions collected were freeze-dried, and the dried fractions were stored at 4 °C until further analysis. All experiments were replicated.

### 2.3. Reagents, Solvents, and Materials

Tris(hydroxymethyl)aminemethane (Tris), 5,5′-dithiobis(2-nitrobenzoic acid) (DTNB), acetylthiocholine iodide (ATCI), S-butyrylthiocholine iodide (BTCI), acetylcholinesterase (AChE) from *Electrophorus electricus*, butyrylcholinesterase (BuChE) from equine serum, bovine serum albumin (BSA), potassium phosphate monobasic (KH_2_PO_4_), potassium phosphate dibasic trihydrate (K_2_HPO_4_·3H_2_O), sodium nitroprusside dihydrate (SNP), sulfanilamide, naphthylethylenediamine dihydrochloride, ortho-phosphoric acid 85%, β-nicotinamide adenine dinucleotide (NADH), phenazine methosulfate (PMS), nitrotetrazolium blue chloride (NBT), 2,2-diphenyl-1-picrylhydrazyl (DPPH), 6-hydroxy-2,5,7,8-tetramethylchroman-2-carboxylic acid (Trolox), 2,2’-azino-bis(3-ethylbenzothiazoline-6-sulfonic acid) (ABTS), potassium persulfate (K_2_O_8_S_2_), sodium carbonate, Folin–Ciocalteau reagent, and gallic acid were purchased from Sigma-Aldrich (St. Louis, MO, USA and Steinheim, Germany). Magnesium chloride hexahydrate and sodium chloride were obtained from VWR (Leuven, Belgium).

The 21 pesticide standards (purity ≥ 95%, Supplementary Table S1) and the internal standards (4,4′-dichlorobenzophenone and triphenyl phosphate) were purchased from Sigma-Aldrich Co. (Darmstadt, Germany). Standard solutions of 14 organochlorine pesticides (α-, β-, γ-, and δ-hexachlorocyclohexanes (HCHs), hexachlorobenzene (HCB), o,p′-DDT ([1,1,1 trichloro-2, 2-bis-(p-chlorophenyl) ethane]), p,p′-DDE ([2,2bis(p-chlorophenyl)-1,1-dichloroethylene]), p,p′-DDD (dichlorodiphenyldichloro-ethane), aldrin, dieldrin, endrin, α, β-endosulfan, and methoxychlor) and 7 organophosphorus pesticides (dimethoate, diazinon, chlorpyrifos-methyl, parathion-methyl, malathion, chlorpyrifos, and chlorfenvinphos) were prepared in n-hexane (Chromatography grade) supplied by Merck (Steinheim, Germany). For the solid-phase extraction (SPE), C18e (500 mg/3 mL) solid-phase extraction (SPE) cartridges were provided by Phenomenex (Madrid, Spain), and methanol was supplied by Sigma-Aldrich (Steinheim, Germany).

A total of 30 compounds (Supplementary Table S1) embracing 14 pharmaceuticals used in Alzheimer’s and Parkinson’s diseases, 8 psychiatric drugs, and 8 metabolites were the target of the present study. Pharmaceuticals used in Alzheimer’s and Parkinson’s diseases (amantadine hydrochloride, apomorphine hydrochloride, benserazide hydrochloride, carbidopa, entacapone, R(-)-deprenyl hydrochloride (selegiline hydrochloride), donepezil hydrochloride, galanthamine hydrochloride, pramipexole dihydrochloride monohydrate, safinamide mesylate salt, rasagiline mesylate, rivastigmine hydrogen tartrate, ropinirole hydrochloride, and rotigotine hydrochloride) were acquired from Sigma-Aldrich (Madrid, Spain), diazepam was purchased from Lipomed AG (Arlesheim, Switzerland), the metabolites of citalopram (citalopram N-oxide hydrochloride, citalopram propionic acid, demethylcitalopram hydrochloride, and didemethylcitalopram hydrochloride) were purchased from H. Lundbeck (Copenhagen, Denmark), norsertraline hydrochloride (sertraline metabolite) was obtained from Cerilliant-Certified Reference Materials (Round Rock, TX, USA), and the remaining compounds (carbamazepine, citalopram, O-desmethylvenlafaxine (venlafaxine metabolite), 10,11-epoxy carbamazepine (carbamazepine metabolite), fluoxetine hydrochloride, norfluoxetine hydrochloride (fluoxetine metabolite), paroxetine hydrochloride, sertraline hydrochloride, trazodone hydrochloride, and venlafaxine hydrochloride) were acquired from Sigma-Aldrich (Madrid, Spain). Carbamazepine-d10 and venlafaxine-d6 purchased as a methanolic solution (Cerilliant-Certified Reference Materials, Round Rock, TX, USA), fluoxetine-d5 hydrochloride prepared in methanol (Sigma-Aldrich, Madrid, Spain), and diazepam-d5 purchased as a methanolic solution (Lipomed AG, Arlesheim, Switzerland) were used as isotopically labeled internal standards (ILIS) in the positive ionization mode, and ibuprofen-d3 purchased as a methanolic solution (Sigma-Aldrich, Madrid, Spain) was used as ILIS in the negative ionization mode. Individual stock standards were prepared at a concentration of 1 g/L on a weight basis. Psychiatric drugs and metabolites were prepared in methanol, and norsertraline hydrochloride was purchased as a methanolic solution. Rotigotine and entacapone were prepared with ethanol, carbidopa was prepared in methanol, donepezil, ropinirole, amantadine, benserazide, rasagiline, pramipexole, and galanthamine were prepared with a mixture of 66.6% ultrapure water and 33.33% methanol, and selegiline, rivastigmine, apormorfine, and safinamide were prepared with a mixture of 66.6% ultrapure water and 33.33% ethanol.

Methanol MS grade Hipersolv CHROMANORM^®^ was purchased from VWR (Gliwice, Poland), acetonitrile MS grade Hipersolv CHROMANORM^®^ was acquired from VWR (Fontenay-sous-Bois, France), propanol MS grade was obtained from Sigma-Aldrich (Steinheim, Germany), and formic acid (PA-ACS) and ethanol were purchased from Carlo Erba (Rodano, Italy). Chromatographic solvents were filtered through a 0.22 µm nylon membrane (Fioroni Filters, Ingré, France) using a vacuum pump (Dinko D-95, Barcelona, Spain) and degassed for 15 min in an ultrasonic bath (Sonorex Digital 10P, Bandelin DK 255P, Berlin, Germany). Nylon syringe filters (0.22 µm, 13 mm) were used for filter fraction extracts (Specanalitica, Carcavelos, Portugal).

Ultrapure water (resistivity of 18.2 MΩ·cm at 25 °C) was prepared using a Simplicity 185 system (Millipore, Molsheim, France).

### 2.4. Chemical Composition of SWE Fractions

#### 2.4.1. Total Phenolic and Total Phlorotannin Contents

The total phenolic content (TPC) of the four fractions was measured through a colorimetric assay using the Folin–Ciocalteau reagent [41], with gallic acid (GA) as the standard (Abs = 0.00722 × GAE + 0.0651; R^2^ = 0.999). Quantification was performed using 96-well plates in a Synergy HT W/TRF multimode microplate reader (BioTek Instruments, Winooski, VT, USA) using Gen5 2.0 software (BioTek Instruments). The assays were performed in triplicate and the results were expressed as mg of gallic acid equivalents (GAE) per gram extract (dry weight, dw).

The quantification of total phlorotannins (1,3,5-substituted phenols) was based on the reaction with 2,4-dimethoxybenzaldehyde (DMBA), as described previously [42]. Measurements were performed using 96-well plates in a Synergy HT microplate reader using phloroglucinol (Phl) as the standard (Abs = 0.0307 × PhlE + 0.0956; R^2^ = 0.998). The assays were performed in triplicate, and the results were expressed as mg of Phl equivalents per g of extract (dw).

#### 2.4.2. Maillard Reaction Products and Browning Index

Maillard reaction products were assessed at 294, 360, and 420 nm, and the formation of fluorescent glycation end-products (AGEs) was estimated by measuring the fluorescence at a set of excitation/emission wavelengths of 360 ± 40 nm/460 ± 40 nm in a Synergy HT microplate reader, after appropriate dilution of samples [42,43]. The analysis was performed in triplicate.

The color parameters of yellow to yellow-brown and the browning index (Br) were calculated as reported previously [42].

#### 2.4.3. Iodine Determination

The extracts’ total iodine (I) content was assessed using a modified Sandell–Kolthoff reaction described previously [44] using a Synergy HT Microplate Reader. Measurements were performed in triplicate.

#### 2.4.4. Pesticide and Pharmaceutical Analysis

Extractions for the pesticides’ analysis were accomplished using a solid-phase extraction methodology followed by gas chromatography analysis. SWE algae extracts were diluted with ultrapure water (1:1) and passed through the SPE C18e cartridge, and the procedure was performed according to Silva et al. [45]. The 14 organochlorine pesticides were analyzed by gas chromatography/electron capture detection according to the method described by Fernandes et al. [46], and the 7 organophosphorus pesticides by gas chromatography/flame photometric detector [46]. Chromatographic analysis for the target pharmaceuticals was carried out on a Shimadzu Nexera UHPLC system (LCMS-8030, Shimadzu Corporation, Kyoto, Japan) coupled to a triple-quadrupole mass spectrometer and operated in the electrospray ionization mode. Lab Solutions software (Shimadzu Corporation, Kyoto, Japan) was used for control and data processing. The mass spectrometer was operated in multiple reaction monitoring mode (MRM). Argon was used as the collision-induced dissociation gas at a pressure of 230 kPa, and nitrogen was used as a nebulizing and drying gas. All the pharmaceuticals were analyzed in the positive ionization mode except for entacapone and citalopram propionic acid, which were analyzed in the negative ionization mode. Two programs were developed to analyze the studied pharmaceuticals and their metabolites. A CortecsTM UPLC^®^ C18+ column (100 mm × 2.1 mm i.d., 1.6 µm particle size) from Waters (Milford, MA, USA) was used for the chromatographic analysis. Eluent A was 0.1% formic acid in ultrapure water and eluent B was acetonitrile LCMS grade for positive ionization mode. The gradient elution started with 5% of eluent B, increasing to 100% B in 3 min, maintained at 100% B during 0.5 min, and returned to initial conditions within 0.5 min. The column was re-equilibrated for 3 min before the next injection. Eluent A was ultrapure water for negative ionization mode, and eluent B was acetonitrile LCMS grade. The gradient elution started with 10% of eluent B, increasing to 100% B in 5.5 min, maintained at 100% B during 1 min, and returned to initial conditions within 0.5 min. The column was re-equilibrated for 2 min before the next injection. A flow rate of 0.3 mL/min was used in both chromatographic programs, and the injection volume was 5 µL. The column oven was set at 30 °C, and the auto-sampler was operated at 4 °C. The needle was rinsed before and after sample aspiration using acetonitrile:methanol:propanol (1:1:1, *v*/*v*/*v*).

### 2.5. Bioactivities

#### 2.5.1. Radical Scavenging Activities

The antiradical activity of the extracts was evaluated by several complementary in vitro assays, namely 2,2-diphenyl-1-picryl-hydrazyl-hydrate free radical scavenging (DPPH^•^), 2,2′-azino-bis(3-ethylbenzothiazoline-6-sulfonic acid assay (ABTS^•+^), superoxide anion radical scavenging (O_2_^•−^), and nitric oxide radical scavenging (^•^NO), according to established procedures [41,42,47]. For the DPPH^•^ assay, a calibration curve was prepared with Trolox (Abs = −0.00630 × TE + 0.716; R^2^ = 0.998), and the antioxidant activity was expressed as mg of Trolox equivalents per g of dw of extract (mg TE/g dw). In ABTS^•+^, the absorbance was taken at 734 nm, and TE was also used as the standard (Abs = −0.00415 × TE + 0.673; R^2^ = 0.995). The obtained results were expressed as mg of TE equivalents per g of dw of extract (mg TE/g dw). For all the assays, triplicate measurements were made for each extract. Concerning O_2_^•−^ and ^•^NO scavenging activities, results are expressed as IC_50_ values and samples were tested in triplicate, and the experiments were repeated three times.

#### 2.5.2. Cholinesterase Inhibition

AChE and BuChE inhibition assays were performed according to the procedure described by Soares et al. [42], based on the quantification of 5-thio-2-nitrobenzoic acid (TNB) production. Results are expressed as IC_50_ values. Samples were tested in triplicate, and the experiments were repeated three times.

### 2.6. Statistical Analysis

All results are reported as mean ± SD or mean ± SEM. The IC_50_ values were calculated using GraphPad Prism Software, version 8. One-way analysis of variance (ANOVA) with Tukey’s as a post hoc test (for comparison of more than three samples) or *t*-test (comparison of two samples) were used to evaluate the differences between the four fractions in terms of IC_50_ values and compound content (GraphPad Prism Software, version 8, San Diego, CA, USA). Differences at *p* < 0.05 were considered statistically significant.

## 3. Results and Discussion

### 3.1. SWE Composition

#### 3.1.1. Total Phenolic Content and Total Phlorotannin Content

The total phenolic and phlorotannin contents of *Himanthalia elongata* (L.) S.F.Gray and *Eisenia bicyclis* (Kjellman) Setchell SWE fractions 1 to 4 are shown in Table 1.

While no trend was observed between TPC and temperature for *E. bicyclis* fractions, higher SWE extraction temperatures led to a higher phenolic yield in the case of *H. elongata*. This result generally agrees with that described in the literature, for instance, for white wine grape pomace [40], microalgae (*Chlorella vulgaris* Beijerinck), macroalgae (*Sargassum vulgare* C.Agardh, *Sargassum muticum* (Yendo) Fensholt, *Porphyra* spp., Cystoseira abies-marina (S.G.Gmelin) C.Agardh, *Undaria pinnatifida* (Harvey) Suringar, *Halopitys incurvus* (Hudson) Batters), and medicinal and aromatic plants (*Rosmarinus officinalis* L., *Thymus vulgaris* L., and *Verbena officinalis* L.) [43].

To the best of our knowledge, there are no previous reports on the TPC value of SWE of these two seaweed species.

Nonetheless, Cofrades et al. [48] showed that the TPC value in 50% aqueous methanolic extract of *H. elongata* was particularly high, 23.47 g GAE/100 g dw, in comparison with brown alga *Undaria pinnatifida* (Harvey) Suringar (4.46 g GAE/100 g dw) and red alga *Porphyra umbilicalis* (L.) J.Agardh (5.53 g GAE/100 g dw). Moreover, Rajauria et al. [5] tested different *H. elongata* extracts (aqueous, methanolic, and hydromethanolic (20–80%)) and reported TPC values ranging from 59.8 mgGAE/g (methanolic extract) to 286.0 mgGAE/g (60% methanol), while the aqueous extract contained 116.5 mgGAE/g. TPC values between 6.60 and 162.22 mgGAE/g were also obtained for *H. elongata* by Silva et al. [49] for different organic extracts. Concerning *E. bicyclis*, Kown et al. [6] evaluated different fractions, obtaining TPC values in the following order: ethyl acetate fraction (263.27 mgGAE/g) > butanol fraction (169.79 mgGAE/g) > hexane fraction (56.12 mgGAE/g) > chloroform fraction (47.86 mgGAE/g) > water fraction (15.90 mgGAE/g).

Regarding TPhC, higher contents were found in fractions 1 and 2 for both seaweeds, corresponding to 8.5% and 3.9% of the total TPC in fraction 1 for *H. elongata* and *E. bicyclis*, respectively. Heffernan et al. [50] obtained a phlorotannin content of 198.28 ± 9.17 (μg PE/mg sample) in ethanol/water extracts of *H. elongata*. Kim et al. [51] investigated the seasonal variation of the phlorotannins content during the lifecycle of *E. bicyclis* using 100% ethanol at room temperature for 12 h. These authors reported extraction yields between 2.13% and 0.56% of the fresh weight in July until the cold season (December–April), respectively.

Nonetheless, it is worth noting that, during the extraction procedure, some components initially present in the sample can be released and may react, producing new compounds. One such chemical event is the Maillard reaction. Plaza et al. [43] showed that this interaction occurs during SWE of natural samples (including seaweeds) at high temperatures, positively affecting the overall antioxidant capacity of the samples and forming products prone to react with the F-C reagent, leading to an overestimation of the TPC. In addition, the increase of the ionic product of water at the temperature/pressure conditions used in SWE makes the water more reactive and able to generate new biologically active compounds through Maillard reactions.

#### 3.1.2. Maillard Reaction Products

**Figure 1:**
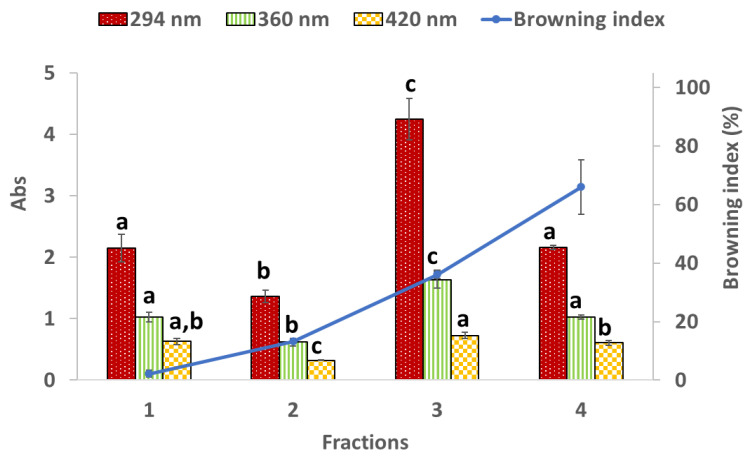
Maillard reaction products and browning index for *Himanthalia elongata* (L.) S.F.Gray. Results are expressed as mean ± SD of three experiments (*n* = 3). For each wavelength, different superscript letters mean statistically significant differences (*p* < 0.05).

**Figure 2:**
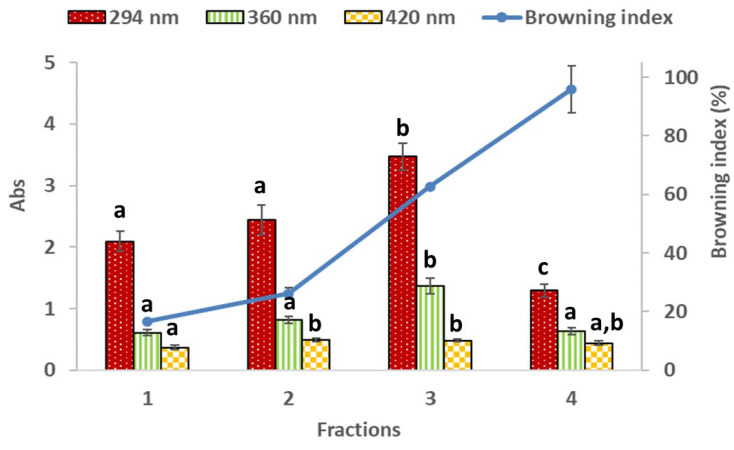
Maillard reaction products and browning index for *Eisenia bicyclis* (Kjellman) Setchell. Results are expressed as mean ± SD of three experiments (*n* = 3). For each wavelength, different superscript letters mean statistically significant differences (*p* < 0.05).

Intermediate colorless Maillard reaction products are usually detected by UV absorbance at 294 nm, while the final stage compounds at 360 and 420 nm [42]. The intermediate compounds are considered precursors of the browning products in the Maillard reaction or caramelization, and in the final stage, melanoidins or heterocyclic compounds, also known as advanced glycation end-products (AGEs), are produced from active intermediate products [42,52]. Figure 1 and Figure 2 show that the absorbance recorded at 294 nm increased from fraction 1 to fraction 3 for both seaweeds, and decreased in fraction 4. Regarding absorbances at 360 and 420 nm, temperature favored the formation of the final stage products since absorbances at 360 and 420 nm increased mainly for *H. elongata*. These results agree with the browning index obtained (blue line in Figure 1 and Figure 2). The browning index increased (*p* < 0.05) in every fraction of the extracts for both seaweeds.

The fluorescent advanced glycation end-products (AGEs) or melanoidins, other Maillard reaction products, have strong light emission between 400 and 500 nm upon excitation at 360 or 370 nm [42]. Overall, the fluorescent AGEs content increased with temperature (Table 2) until fraction 3 (190 °C) for both seaweeds, with fraction 4 showing a decrease in its values for *H. elongata* and *E. bicyclis*. This indicates that higher formation of intermediate and final Maillard reaction products was obtained in stage 3 (at 190 °C) for both seaweeds.

Comparing the Maillard reaction products and the TPC content of seaweeds, it can be observed that, particularly for *E. bicyclis*, there is an increase in the TPC content in fraction 3 that can be positively related to AGEs present in this fraction (Table 2) [43]. According to Plaza et al. [43] and Grigoriou and Pinakoulaki [53], besides phenolics, there are other classes of compounds that positively react with Folin–Ciocalteu reagent due to the presence of reducing groups. One example is the Maillard reaction products. As shown, this class of compounds are present in higher amounts in the fractions obtained at higher temperatures (190 and 250 °C) than at lower temperatures (90 and 140 °C), thus contributing to increased TPC values. Regarding the DPPH^•^ and ABTS^•+^ scavenging activities (see Section 3.2.1), these are higher and significantly different for fractions 3 and 4 compared with fractions 1 and 2. Again, these results suggest a positive relation between Maillard reaction products and the scavenging activities of the extracts. Maillard reaction products are very complex compounds, and their antioxidant activity can include reducing power ability, scavenging of free radicals, metal ion chelating activity, and regulation of intracellular antioxidant enzymes in vivo [52]. The antioxidant mechanisms of melanoidins were attributed to the radical scavenging activity and the metal chelating capacity resulting from its anionic hydrophilic nature that can form stable complexes with metal cations [52].

#### 3.1.3. Iodine

Seaweeds are a rich source of essential elements, and iodine (I), in particular, is very abundant, with reported values between 4.3 and 2660 mg/kg wet weight [54,55]. The well-known health benefits of I are associated with its role in the functioning of the thyroid gland and the associated production of thyroid hormones [56,57]. The World Health Organization recommends an I daily intake (RDI) of 150 μg and a tolerable upper intake level (UL) of 600 μg/day [58], considering that ingestion at levels above the RDI can also negatively impact human health [57]. Considering the potential health applications of the SWE extracts, it is crucial to assess the I amount present in each fraction according to the WHO recommendation. The levels of iodine measured in *H. elongata* in the four fractions were 92.6 ± 5.2, 79.8 ± 1.5, 35.6 ± 5.0, and 12.5 ± 0.7 µg/g extract dw for fractions 1 to 4, respectively. Regarding *E. bicyclis*, the values found were 635 ± 42, 134 ± 11, 98.7 ± 8.1, and 7.51 ± 0.85 µg/g extract dw for fractions 1 to 4, respectively. Both seaweeds presented the same trend, with a higher iodine content in fraction 1 and the lowest for fraction 4, decreasing with the temperature increase (from 90 °C in fraction 1 to 250 °C in fraction 4). No studies about SWE effects on iodine content for *H. elongata* and *E. bicyclis* were found in the literature. However, Soares et al. [42] assessed the influence of SWE using different extraction temperatures (90 to 250 °C) on the recovery of several compounds, including iodine, from the green alga *Codium tomentosum* Stackhouse and brown seaweed *Fucus vesiculosus* L. These authors reported that the amount of iodine extracted from both seaweeds was higher in fractions obtained at a lower temperature (90 °C) and lower for the fractions obtained at higher temperatures (250 °C). The results imply that iodine seems to be present in its inorganic form, easily extractable with water [42,54], and the extraction steps at lower temperatures (90 and 140 °C) are the ones with the highest yield. Iodine values reported in the literature for *H. elongata* were 116.6 ± 22.62 [59] and 135 ± 21 µg/g dw [60] of seaweed. Regarding *E. bicyclis*, 600 and 586 ± 56 µg of I/g dw of seaweed were reported [61,62]. Several factors are responsible for different values when measuring I in seaweeds, such as the growth stage, sampled algal tissue, sampling localization, salinity, tidal amplitude, processing, harvest conditions, and temperature [54]. Considering the WHO recommendations, fraction 1 of *E. bicyclis* should be used with caution.

#### 3.1.4. Analysis of Pesticides and Pharmaceuticals in SWE Seaweed Extracts

To ensure that the 8 fractions obtained after SWE extraction (4 of *H. elongata* and 4 of *E. bicyclis*) were pesticide-free, they were analyzed by the proposed SPE/GC-ECD and SPE/GC-FPD methodology [45,46]. The 21 target pesticides (14 organochlorine and 7 organophosphorus pesticides) were not detected in the SWE extracts. However, organochlorine pesticides have recently been reported [63], and bioaccumulation of these pesticides in algae has also been observed [10]. Regarding organophosphorus pesticides, García-Rodríguez et al. [64], despite detecting trace amounts of other pesticide families, did not detect organophosphorus pesticides in seaweeds. Pharmaceuticals and metabolites were also analyzed in the seaweed SWE fractions, but none of the 30 target compounds were observed. In 2021, Ojemaye et al. [65] reported the analysis of six pharmaceuticals (acetaminophen, sulfamethoxazole, diclofenac, carbamazepine, triclosan, and lamivudine) and one stimulant (caffeine) in five seaweed species (*Ulva* sp., *Gelidium pristoides* (Turner) Kützing, *Bifurcaria brassicaeformis* (Kützing) E.S.Barton, *Caulerpa filiformis* (Suhr) K. Hering, and *Aeodes orbitosa* (Suhr) F.Schmitz). Acetaminophen, sulfamethoxazole, diclofenac, lamivudine, and carbamazepine were detected in all samples. For most of the analyzed samples, diclofenac was the pharmaceutical detected with the highest frequency and concentration. The study conducted in 2018 by Helou et al. [66] mentioned the detection of two illicit drugs (cocaine and methadone) and two behavioral medicines (carbamazepine and diazepam) in edible seaweeds. In 2021, Soares et al. [42] published a study in which 115 compounds, embracing 82 pharmaceuticals (non-steroidal anti-inflammatory drugs, analgesics, antibiotics, anorexics, anxiolytics, beta-blockers, laxatives, stimulants, and psychiatric drugs) and 33 polar pesticides, were screened in the SWE fractions of *F. vesiculosus* and *C. tomentosum*. None of these pollutants were detected [42]. To the best of our knowledge, no studies evaluating the presence of organochlorine and organophosphorus pesticides and pharmaceuticals used for Alzheimer’s and Parkinson’s diseases treatment in SWE seaweed extracts were published. In the present work, the compounds were screened on the SWE fractions and not on the seaweed samples. Therefore, the obtained results suggested that the SWE fractions are safe concerning the analyzed target compounds for a potential application.

### 3.2. Bioactivities

#### 3.2.1. Antioxidant Activity

The DPPH^•^ radical scavenging potential of the SWE *H. elongata* and *E. bicyclis* fractions is shown in Table 3, according to the calibration curve obtained for Trolox.

The obtained values for TE show that higher extraction temperatures present an increased DPPH*^•^* scavenging activity. However, it is worth mentioning that, for *H. elongata*, a significant gap was observed between the first two and the last two fractions, and the TE value for fraction 2 was five times lower than the one assessed for fraction 3. Furthermore, there was no statistical difference between the third and fourth fractions for both seaweed species. Silva et al. [49] evaluated the ability of different extracts (ethanolic, acetone, ethyl acetate, hexane, and chloroform) of *H. elongata* to scavenge DPPH^•^. They observed that the hexane extract was the most active (75.33 mgTE/g), and at the same time, contained the lowest amount of phenolics (6.60 mgGAE/g), reinforcing the idea that other compounds may also be involved in the overall antioxidant activity of seaweeds.

Kwon et al. [6] tested different *E. bicyclis* fractions against DPPH^•^, observing that ethyl acetate was the most active, followed by butanol and hexane fractions. Water and chloroform fractions were not active. The results obtained by these authors demonstrated that DPPH^•^ scavenging and TPC values were positively correlated, which was not verified in the current study.

Table 3 displays the results of ABTS^•+^ radical scavenging potential of the SWE *H. elongata* and *E. bicyclis* fractions. As seen thus far, higher extraction temperatures continue to yield higher antioxidant activity. Moreover, there was no significant difference between the TE values for the first and second fractions. The considerable gap in TE values between the first two and the last two fractions of *H. elongata* is also worth noting, similarly to the results observed for the DPPH^•^ scavenging assay. In the case of *E. bicyclis*, higher temperatures also favored ABTS^•+^ scavenging activity, although fraction 3 was more active than fraction 4.

^●^NO and O_2_^●−^ radicals have important physiological roles as vascular signaling molecules. However, when overproduced, they react, generating the highly cytotoxic ONOO^−^. An imbalance between these ROS and RNS and the endogenous antioxidant system leads to oxidative and nitrosative stresses, two pathways involved in neurodegeneration progression [67].

All fractions showed ^•^NO scavenging activity, inhibiting nitrite production up to about 70%, with concentrations of 0.5 mg/mL extracts and upwards. Nonetheless, fractions extracted with higher temperatures presented a lower half-maximal inhibitory concentration (IC_50_), as seen in Table 3, with fraction 4 requiring 0.246 mg/mL of *H. elongata* and 0.316 mg/mL of *E. bicyclis* to prevent 50% of the nitric oxide radicals from reacting with oxygen. Nonetheless, these IC_50_ values are not statistically different from those obtained for fractions 1, 2, and 3.

Once again, all extracts showed strong antioxidant activity, scavenging 100% of O_2_^•−^ with concentrations of 0.5 mg/mL extracts and upwards. However, as seen for ^•^NO scavenging, fractions extracted with higher temperatures presented lower IC_50_ values, as summarized in Table 3. Fraction 4 notably displayed the most promising results, needing only 0.0530 mg/mL (for *H. elongata*) to prevent 50% of the superoxide anion radicals from reacting with the NBT reagent, a quarter of the 0.203 mg/mL needed of fraction 1. A similar trend was found for *E. bicyclis* fractions, although fraction 4 of *E. bicyclis* was less active than the corresponding one of *H. elongata*.

Although no information was found in the literature regarding the IC_50_ for *H. elongata*’s ^•^NO and O_2_^•−^ scavenging activity, Soares et al. [42] conducted a study on green alga *C. tomentosum* and brown seaweed *F. vesiculosus* fractions obtained through SWE. This research found that the fractions extracted at 250 °C were the most active overall, and the brown alga was more active than *C. tomentosum* regarding ^•^NO scavenging activity, presenting an IC_50_ of 132.4 and 254.2 μg/mL, respectively. On the other hand, the green alga showed the lowest IC_50_ value for O_2_^•−^ scavenging activity, 85.7 μg/mL. However, even though *H. elongata* is also a brown seaweed, its IC_50_ values were closer to *C. tomentosum*. It is also worth noting that *H. elongata* was the most active regarding O_2_^•−^ scavenging out of all three algae mentioned, achieving the lowest IC_50_ value of 0.0530 mg/mL [68,69].

In general, the SWE *H. elongata* and *E. bicyclis* extracts showed great antioxidant activity, especially regarding the inhibition of the O_2_^•−^. These results are generally consistent with those described in the literature, as brown algae have been shown to possess great radical scavenging activity (including ROS, RNS, DPPH^•^, and ABTS^•+^), both in noncellular systems and ex vivo, due to their characteristic high amounts of phenolic compounds and carotenoids, such as fucoxanthin [70].

Moreover, it has been observed that the extraction temperature plays a vital role in the antioxidant capacity of the samples. This is also in agreement with the literature and may further suggest that the compounds responsible for *H. elongata* and *E. bicyclis* antioxidant properties are mostly less polar since studies have shown that the extraction ability of subcritical water toward the more polar compounds decreases with the increase in temperature [71,72]. Nonetheless, further research is needed to confirm the nature and identify the specific bioactive compounds present in *H. elongata*.

These results are also consistent with the outcome observed for the TPC assay and the phlorotannin content, suggesting that the algal polyphenols such as phlorotannins present in fractions 1 and 2 could indeed be the primary constituents responsible for the antiradical properties of the extracts of these fractions [5]. Several studies have linked *H. elongata* antioxidant capacity to the seaweed’s high TPC [48,73,74,75]. Regarding fractions 3 and 4, the phlorotannin content is <LOD, but the Maillard reaction products are present in high quantities (Table 2), possibly being responsible for the antiradical properties obtained. Several authors have already reported the high bioactivities (antioxidant and neuroprotective) of the Maillard reaction products formed during food processing at high temperatures and the positive correlation with browning development [52,76].

#### 3.2.2. AChE and BuChE Inhibition

None of the fractions studied showed significant cholinesterase inhibition, not able to suppress enzyme activity over 50%. Fraction 4 was the most promising in both cases, presenting about 40% and 50% AChE inhibition at 2 mg/mL for *H. elongata* and *E. bicyclis*, respectively. For BuChE, both fractions 4 displayed ca. 40% inhibition (Table 4). Due to solubility problems, higher concentrations of this fraction could not be further studied to assess its inhibitory potential.

Once again, it was challenging to find comparable information regarding *H. elongata*’s anti-ChE activity in the literature. Nonetheless, a previous study on another brown alga, *F. vesiculosus*, has shown that fractions obtained through SWE also presented no AChE or BuChE inhibition over 50%. Furthermore, as seen in the present study, only the fraction extracted at the highest temperature achieved enzyme inhibition close to 40% [42]. However, André et al. [77] reported anti-AChE activity up to 90% on aqueous extracts (decocted at 100 °C for 30 min) of three different *F. vesiculosus* samples. Choi et al. [78] also showed that ethanolic extracts of brown alga *E. bicyclis* possessed potent AChE and BuChE inhibition activity, particularly 68.01% ± 1.37% and 95.72% ± 3.80% at 25 µg/mL, with IC_50_ values of 2.78 ± 0.07 and 3.48 ± 0.32 μg/mL, respectively. However, their extract was much more active than our fractions.

These results suggest that the extraction method plays an important role in the anti-ChE activity of algal samples, and SWE may negatively affect this inhibitory capacity. Therefore, further research is required to confirm this conclusion.

## 4. Conclusions

SWE is a green extraction process proven to be a very efficient technique for obtaining highly bioactive fractions. These fractions are considered safe regarding a total of 51 contaminants (21 pesticides and 30 pharmaceuticals) screened, and their iodine content is safe for consumption, except fraction 1 of *Eisenia bicyclis* (Kjellman) Setchell, that should be used with caution.

In this study on *Himanthalia elongata* (L.) S.F.Gray and *E. bicyclis*, the samples’ antioxidant capacity, the total phenolic content, and the Maillard reaction products depended on the extraction temperature. Furthermore, they varied among the fractions extracted, with the third and fourth fractions showing the most promising results.

Overall, even though no significant ChE inhibition was detected (below 50%), fractions 3 and 4 were the ones with the highest biological activities, namely DPPH^•^, ABTS^•+^, ^•^NO, and O_2_^•−^ scavenging activity, which can be tentatively explained due to the presence of Maillard reaction products.

Future research should focus on ascertaining *H. elongata*’s and *E. bicyclis’* ability to inhibit other brain enzymes associated with neurodegenerative etiology, such as tyrosinase and monoamine oxidase A and B.

Lastly, HPLC analysis could provide important insight into the composition of *H. elongata* and *E. bicyclis* fractions and the specific compounds responsible for their high antioxidant capacity.

## Figures and Tables

**Table 1:** Total phenolic content (TPC) and total phlorotannin content (TPhC) of *Himanthalia elongata* (L.) S.F.Gray and *Eisenia bicyclis* (Kjellman) Setchell fractions.

Seaweed	Fraction	TPC	TPhC
		(mg GAE/g Extract dw)	(mg Phl/g Extract dw)
*H. elongata*	1	5.80 ± 0.08 ^a^	0.498 ± 0.016 ^a^
	2	10.7 ± 0.5 ^b^	0.133 ± 0.009 ^b^
	3	53.0 ± 0.5 ^c^	<LOD
	4	71.1 ± 2.5 ^d^	<LOD
*E. bicyclis*	1	33.4 ± 2.5 ^a,c^	1.29 ± 0.06 ^a^
	2	20.6 ± 1.2 ^a^	1.20 ± 0.05 ^b^
	3	55.4 ± 13.3 ^b^	0.114 ± 0.014 ^c^
	4	44.7 ± 3.9 ^c^	<LOD

dw: dry weight; LOD: limit of detection. Results are expressed as mean ± SD of three determinations. Different superscript letters within each seaweed represent significant differences at *p* < 0.05.

**Table 2:** Advanced glycation end-products (AGEs) formed during the Maillard reaction for *Himanthalia elongata* (L.) S.F.Gray and *Eisenia bicyclis* (Kjellman) Setchell.

Seaweed	Fraction	AGEs
*H. elongata*	1	280 ± 28 ^a^
	2	335 ± 35 ^a^
	3	2915 ± 191 ^b^
	4	1570 ± 99 ^c^
*E. bicyclis*	1	67.7 ± 2.5 ^a^
	2	225 ± 21 ^a^
	3	4400 ± 283 ^b^
	4	1315 ± 78 ^c^

Results are expressed as mean ± SD of three determinations. Different superscript letters within each seaweed represent significant differences at *p* < 0.05.

**Table 3:** Scavenging activity of *Himanthalia elongata* (L.) S.F.Gray and *Eisenia bicyclis* (Kjellman) Setchell fractions.

Seaweed	Fraction	DPPH (mg TE/g Extract dw)	ABTS^•+^ (mg TE/g Extract dw)	^•^NO (IC_50_, mg/mL)	O_2_^•−^ (IC_50_, mg/mL)
*H. elongata*	1	2.62 ± 2.34 ^a^	14.9 ±z 0.4 ^a^	0.379 ^a^	0.203 ^a^
	2	6.06 ± 0.09 ^b^	20.1 ± 2.0 ^a^	0.316 ^a^	0.120 ^b^
	3	30.2 ± 0.9 ^c^	123 ± 8 ^b^	0.313 ^a^	0.119 ^b^
	4	28.3 ± 0.5 ^c^	140 ± 3 ^c^	0.246 ^a^	0.0530 ^c^
*E. bicyclis*	1	26.8 ± 1.8 ^a^	34.0 ± 0.8 ^a^	0.257 ^a^	0.393 ^a^
	2	38.8 ± 5.5 ^a,b^	32.5 ± 2.6 ^a^	0.340 ^a^	0.336 ^a^
	3	54.1 ± 12.0 ^b^	112 ± 20 ^b^	0.486 ^b^	0.192 ^b^
	4	52.7 ± 3.9 ^b^	69.1 ± 12.0 ^c^	0.308 ^a^	0.173 ^b^

dw: dry weight. Results are expressed as mean ± SD of three determinations or IC_50_ values. Different superscript letters within each seaweed represent significant differences at *p* < 0.05.

**Table 4:** AChE and BuChE inhibition (%) of SWE of *Himanthalia elongata* (L.) S.F.Gray and *Eisenia bicyclis* (Kjellman) Setchell.

		AChE Inhibition (%, Mean ± SEM)				BuChE Inhibition (%, Mean ± SEM)			
Seaweed	Concentration (mg/mL)	1	2	3	4	1	2	3	4
*H. elongata*	2.00	n.a.	n.a.	n.a.	35.8 ± 11.2 ^a^	n.a.	n.a.	20.2 ± 7.3 ^a^	39.3 ± 7.9 ^a^
	1.00	n.a.	n.a.	n.a.	24.2 ± 3.6 ^a^	n.a.	n.a.	n.a.	27.0 ± 11.6 ^a^
*E. bicyclis*	2.00	n.a.	n.a.	33.7 ± 6.9 ^a^	49.3 ± 0.97 ^a^	n.a.	n.a.	29.6 ± 7.2 ^a^	37.8 ± 8.6 ^a^
	1.00	n.a.	n.a.	24.3 ± 3.7 ^a^	35.3 ± 6.6 ^a^	n.a.	n.a.	24.5 ± 1.6 ^a^	26.9 ± 3.9 ^a^

n.a.—not active, % of inhibition below 10%. Results are expressed as mean ± SEM of three independent assays, each one performed in triplicate. The same superscript letters in the same row correspond to non-statistically significant differences (*p* > 0.05).

## Supplementary Materials

The following supporting information can be downloaded at: https://www.mdpi.com/article/10.3390/foods11050746/s1, Table S1: Chemical names, molecular formulas, CAS numbers, and molecular weight of the studied pollutants.

## References

[1] Guiry M.D.R. The Seaweed Site: Information on Marine Macroalgae. http://www.seaweed.ie/index.php.

[2] Pangestuti R., Kim S.K. (2011). Neuroprotective effects of marine algae. Mar. Drugs.

[3] Goecke F., Klemetsdal G., Ergon Å. (2020). Cultivar Development of Kelps for Commercial Cultivation—Past Lessons and Future Prospects. Front. Mar. Sci..

[4] Pinteus S., Silva J., Alves C., Horta A., Fino N., Rodrigues A.I., Mendes S., Pedrosa R. (2017). Cytoprotective effect of seaweeds with high antioxidant activity from the Peniche coast (Portugal). Food Chem..

[5] Rajauria G., Jaiswal A.K., Abu-Gannam N., Gupta S. (2013). Antimicrobial, antioxidant and free radical-scavenging capacity of brown seaweed himanthalia elongata from western coast of Ireland. J. Food Biochem..

[6] Kwon T.H., Kim T.W., Kim C.G., Park N.H. (2013). Antioxidant activity of various solvent fractions from edible brown alga, eisenia bicyclis and its active compounds. J. Food Sci..

[7] Van der Spiegel M., Noordam M.Y., van der Fels-Klerx H.J. (2013). Safety of novel protein sources (insects, microalgae, seaweed, duckweed, and rapeseed) and legislative aspects for their application in food and feed production. Compr. Rev. Food Sci. Food Saf..

[8] Banach J.L., Hoek-van den Hil E.F., van der Fels-Klerx H.J. (2020). Food safety hazards in the European seaweed chain. Compr. Rev. Food Sci. Food Saf..

[9] Zhang L., Gionfriddo E., Acquaro V., Pawliszyn J. (2018). Direct immersion solid-phase microextraction analysis of multi-class contaminants in edible seaweeds by gas chromatography-mass spectrometry. Anal. Chim. Acta.

[10] Qiu Y.W., Zeng E.Y., Qiu H., Yu K., Cai S. (2017). Bioconcentration of polybrominated diphenyl ethers and organochlorine pesticides in algae is an important contaminant route to higher trophic levels. Sci. Total Environ..

[11] Leong W.-H., Teh S.-Y., Hossain M.M., Nadarajaw T., Zabidi-Hussin Z., Chin S.-Y., Lai K.-S., Lim S.-H.E. (2020). Application, monitoring and adverse effects in pesticide use: The importance of reinforcement of Good Agricultural Practices (GAPs). J. Environ. Manag..

[12] Khan A.H., Aziz H.A., Khan N.A., Hasan M.A., Ahmed S., Farooqi I.H., Dhingra A., Vambol V., Changani F., Yousefi M. (2022). Impact, disease outbreak and the eco-hazards associated with pharmaceutical residues: A Critical review. Int. J. Environ. Sci. Technol..

[13] Paíga P., Santos L.H.M.L.M., Ramos S., Jorge S., Silva J.G., Delerue-Matos C. (2016). Presence of pharmaceuticals in the Lis river (Portugal): Sources, fate and seasonal variation. Sci. Total Environ..

[14] Lolić A., Paíga P., Santos L.H.M.L.M., Ramos S., Correia M., Delerue-Matos C. (2015). Assessment of non-steroidal anti-inflammatory and analgesic pharmaceuticals in seawaters of North of Portugal: Occurrence and environmental risk. Sci. Total Environ..

[15] Sánchez-González S., Pose-Juan E., Herrero-Hernández E., Álvarez-Martín A., Sánchez-Martín M.J., Rodríguez-Cruz S. (2013). Pesticide residues in groundwaters and soils of agricultural areas in the Águeda River Basin from Spain and Portugal. Int. J. Environ. Anal. Chem..

[16] Liu N., Jin X., Yan Z., Luo Y., Feng C., Fu Z., Tang Z., Wu F., Giesy J.P. (2020). Occurrence and multiple-level ecological risk assessment of pharmaceuticals and personal care products (PPCPs) in two shallow lakes of China. Environ. Sci. Eur..

[17] Pereira A., Silva L., Laranjeiro C., Pena A. (2021). Assessment of human pharmaceuticals in drinking water catchments, tap and drinking fountain waters. Appl. Sci..

[18] Battaglin W.A., Bradley P.M., Iwanowicz L., Journey C.A., Walsh H.L., Blazer V.S. (2018). Pharmaceuticals, hormones, pesticides, and other bioactive contaminants in water, sediment, and tissue from Rocky Mountain National Park, 2012–2013. Sci. Total Environ..

[19] European Comission (2005). Regulation (EC) No 396/2005 of the European Parliament and of the Council of 23 February 2005 on maximum residue levels of pesticides in or on food and feed of plant and animal origin and amending Council Directive 91/414/EEC (Text with EEA relevance). Off. J. Eur. Union.

[20] Okuzumi J., Nishino H., Murakoshi M., Iwashima A., Tanaka Y., Yamane T., Fujita T., Takahashi T. (1990). Inhibitory effects of fucoxanthin, a natural carotenoid, on N-myc expression and cell cycle progression in human malignant tumor cells. Cancer Lett..

[21] Hrelia P., Sita G., Ziche M., Ristori E., Marino A., Cordaro M., Molteni R., Spero V., Malaguti M., Morroni F. (2020). Common Protective Strategies in Neurodegenerative Disease: Focusing on Risk Factors to Target the Cellular Redox System. Oxid. Med. Cell. Longev..

[22] De Los Ríos C. (2012). Cholinesterase inhibitors: A patent review (2007–2011). Expert Opin. Ther. Pat..

[23] Paudel P., Seong S.H., Zhou Y., Park H.J., Jung H.A., Choi J.S. (2019). Anti-Alzheimer’s Disease Activity of Bromophenolsfrom a Red Alga, Symphyocladia latiuscula (Harvey) Yamada. ACS Omega.

[24] Cornish M.L., Critchley A.T., Mouritsen O.G. (2017). Consumption of seaweeds and the human brain. J. Appl. Phycol..

[25] Dewapriya P., Kim S.K. (2015). Marine Algae for Protecting Your Brain: Neuroprotective Potentials of Marine Algae. Mar. Algae Extr. Process. Prod. Appl..

[26] Teixeira D., Fernandes R., Prudêncio C., Vieira M. (2016). 3-Nitrotyrosine quantification methods: Current concepts and future challenges. Biochimie.

[27] Tsang A.H.K., Chung K.K.K. (2009). Oxidative and nitrosative stress in Parkinson’s disease. Biochim. Biophys. Acta Mol. Basis Dis..

[28] Singh A., Tripathi P., Yadawa A.K., Singh S. (2020). Promising Polyphenols in Parkinson’s Disease Therapeutics. Neurochem. Res..

[29] Ruskovska T., Maksimova V., Milenkovic D. (2020). Polyphenols in human nutrition: From the in vitro antioxidant capacity to the beneficial effects on cardiometabolic health and related inter-individual variability—An overview and perspective. Br. J. Nutr..

[30] Grosso C., Valentão P., Ferreres F., Andrade P.B. (2015). Alternative and Efficient Extraction Methods for Marine-Derived Compounds. Mar. Drugs.

[31] Zhang Q.-W., Lin L.-G., Ye W.-C. (2018). Techniques for extraction and isolation of natural products: A comprehensive review. Chin. Med..

[32] Bamba B.S.B., Shi J., Tranchant C.C., Xue S.J., Forney C.F., Lim L.T. (2018). Influence of extraction conditions on ultrasound-assisted recovery of bioactive phenolics from blueberry pomace and their antioxidant activity. Molecules.

[33] Castro-Puyana M., Herrero M., Mendiola J.A., Ibáñez E. (2013). Subcritical water extraction of bioactive components from algae. Funct. Ingred. Algae Foods Nutraceuticals.

[34] Ciko A.M., Jokić S., Šubarić D., Jerković I. (2018). Overview on the application of modern methods for the extraction of bioactive compounds from marine macroalgae. Mar. Drugs.

[35] Dembek M., Bocian S. (2020). Pure water as a mobile phase in liquid chromatography techniques. TrAC Trends Anal. Chem..

[36] Ko M.-J., Nam H.-H., Chung M.-S. (2020). Subcritical water extraction of bioactive compounds from Orostachys japonicus A. Berger (Crassulaceae). Sci. Rep..

[37] Liang X., Fan Q. (2013). Application of Sub-Critical Water Extraction in Pharmaceutical Industry. J. Mater. Sci. Chem. Eng..

[38] Pinto D., Vieira E.F., Peixoto A.F., Freire C., Freitas V., Costa P., Delerue-Matos C., Rodrigues F. (2021). Optimizing the extraction of phenolic antioxidants from chestnut shells by subcritical water extraction using response surface methodology. Food Chem..

[39] Zhang J., Wen C., Zhang H., Duan Y., Ma H. (2020). Recent advances in the extraction of bioactive compounds with subcritical water: A review. Trends Food Sci. Technol..

[40] Pedras B., Salema-Oom M., Sá-Nogueira I., Simões P., Paiva A., Barreiros S. (2017). Valorization of white wine grape pomace through application of subcritical water: Analysis of extraction, hydrolysis, and biological activity of the extracts obtained. J. Supercrit. Fluids.

[41] Barroso M.F., Ramalhosa M.J., Alves R.C., Dias A., Soares C.M.D., Oliva-Teles M.T., Delerue-Matos C. (2016). Total antioxidant capacity of plant infusions: Assessment using electrochemical DNA-based biosensor and spectrophotometric methods. Food Control.

[42] Soares C., Paíga P., Marques M., Neto T., Carvalho A.P., Paiva A., Simões P., Costa L., Bernardo A., Fernández N. (2021). Multi-step subcritical water extracts of fucus vesiculosus l. And codium tomentosum stackhouse: Composition, health-benefits and safety. Processes.

[43] Plaza M., Amigo-Benavent M., del Castillo M.D., Ibáñez E., Herrero M. (2010). Facts about the formation of new antioxidants in natural samples after subcritical water extraction. Food Res. Int..

[44] Soares C., Švarc-Gajić J., Oliva-Teles M.T., Pinto E., Nastić N., Savić S., Almeida A., Delerue-Matos C. (2020). Mineral composition of subcritical water extracts of Saccorhiza polyschides, a brown seaweed used as fertilizer in the North of Portugal. J. Mar. Sci. Eng..

[45] Silva A.M., Lago J.P., Pinto D., Moreira M.M., Grosso C., Fernandes V.C., Delerue-Matos C., Rodrigues F. (2021). Salicornia ramosissima bioactive composition and safety: Eco-friendly extractions approach (microwave-assisted extraction vs. conventional maceration). Appl. Sci..

[46] Fernandes V.C., Freitas M., Pacheco J.P.G., Oliveira J.M., Domingues V.F., Delerue-Matos C. (2018). Magnetic dispersive micro solid-phase extraction and gas chromatography determination of organophosphorus pesticides in strawberries. J. Chromatogr. A.

[47] Cvetanović A., Švarc-Gajić J., Zeković Z., Jerković J., Zengin G., Gašić U., Tešić Ž., Mašković P., Soares C., Barroso M.F. (2019). The influence of the extraction temperature on polyphenolic profiles and bioactivity of chamomile (*Matricaria chamomilla* L.) subcritical water extracts. Food Chem..

[48] Cofrades S., López-Lopez I., Bravo L., Ruiz-Capillas C., Bastida S., Larrea M.T., Jiménez-Colmenero F. (2010). Nutritional and Antioxidant Properties of Different Brown and Red Spanish Edible Seaweeds. Food Sci. Technol. Int..

[49] Silva A., Rodrigues C., Garcia-Oliveira P., Lourenço-Lopes C., Silva S.A., Garcia-Perez P., Carvalho A.P., Domingues V.F., Barroso M.F., Delerue-Matos C. (2021). Screening of bioactive properties in brown algae from the northwest iberian peninsula. Foods.

[50] Heffernan N., Brunton N.P., FitzGerald R.J., Smyth T.J. (2015). Profiling of the molecular weight and structural isomer abundance of macroalgae-derived phlorotannins. Mar. Drugs.

[51] Kim S.M., Kang S.W., Jeon J.S., Jung Y.J., Kim W.R., Kim C.Y., Um B.H. (2013). Determination of major phlorotannins in *Eisenia bicyclis* using hydrophilic interaction chromatography: Seasonal variation and extraction characteristics. Food Chem..

[52] Fu Y., Zhang Y., Soladoye O.P., Aluko R.E. (2020). Maillard reaction products derived from food protein-derived peptides: Insights into flavor and bioactivity. Crit. Rev. Food Sci. Nutr..

[53] Grigoriou A.M., Pinakoulaki E. (2021). Linking the Dynamic Changes in the In Vitro Antioxidant Activity of Carob Kibbles upon Roasting to the Chemical and Structural Changes Revealed by FTIR Spectroscopy. Antioxidants.

[54] Correia H., Soares C., Morais S., Pinto E., Marques A., Nunes M.L., Almeida A., Delerue-Matos C. (2021). Seaweeds rehydration and boiling: Impact on iodine, sodium, potassium, selenium, and total arsenic contents and health benefits for consumption. Food Chem. Toxicol..

[55] Roohinejad S., Koubaa M., Barba F.J., Saljoughian S., Amid M., Greiner R. (2017). Application of seaweeds to develop new food products with enhanced shelf-life, quality and health-related beneficial properties. Food Res. Int..

[56] Lazarus J.H. (2014). Iodine Status in Europe in 2014. Eur. Thyroid J..

[57] Roleda M.Y., Skjermo J., Marfaing H., Jónsdóttir R., Rebours C., Gietl A., Stengel D.B., Nitschke U. (2018). Iodine content in bulk biomass of wild-harvested and cultivated edible seaweeds: Inherent variations determine species-specific daily allowable consumption. Food Chem..

[58] World Health Organization (2007). Assessment of Iodine Deficiency Disorders and Monitoring Their Elimination: A Guide for Programme Managers.

[59] Romarís-Hortas V., García-Sartal C., del Carmen Barciela-Alonso M., Domínguez-González R., Moreda-Piñeiro A., Bermejo-Barrera P. (2011). Bioavailability study using an in-vitro method of iodine and bromine in edible seaweed. Food Chem..

[60] Nitschke U., Stengel D.B. (2015). A new HPLC method for the detection of iodine applied to natural samples of edible seaweeds and commercial seaweed food products. Food Chem..

[61] Van Netten C., Hoption Cann S.A., Morley D.R., van Netten J.P. (2000). Elemental and radioactive analysis of commercially available seaweed. Sci. Total Environ..

[62] Teas J., Pino S., Critchley A., Braverman L.E. (2004). Variability of iodine content in common commercially available edible seaweeds. Thyroid.

[63] Sundhar S., JeyaShakila R., Jeyasekaran G., Shalini R., Arisekar U., Jenishma J.S. (2019). Safety assessment of edible red seaweeds Gracilaria and Gelidiella of Gulf of Mannar in terms of OCP residual contamination. Environ. Nanotechnol. Monit. Manag..

[64] García-Rodríguez D., Cela-Torrijos R., Lorenzo-Ferreira R.A., Carro-Díaz A.M. (2012). Analysis of pesticide residues in seaweeds using matrix solid-phase dispersion and gas chromatography-mass spectrometry detection. Food Chem..

[65] Ojemaye C.Y., Petrik L. (2021). Pharmaceuticals and Personal Care Products in the Marine Environment Around False Bay, Cape Town, South Africa: Occurrence and Risk-Assessment Study. Environ. Toxicol. Chem..

[66] Helou A.M., Keefe M., Mottaleb M.A., Thomson W.J., Mottaleb M.A. (2018). Analysis of illicit drugs and pharmaceuticals in edible seaweeds by liquid chromatography-tandem mass spectrometry. Anal. Methods.

[67] Cenini G., Lloret A., Cascella R. (2019). Oxidative Stress in Neurodegenerative Diseases: From a Mitochondrial Point of View. Oxid. Med. Cell. Longev..

[68] Lee S.H., Eom S.H., Yoon N.Y., Kim M.M., Li Y.X., Ha S.K., Kim S.K. (2016). Fucofuroeckol-A from *Eisenia bicyclis* Inhibits Inflammation in Lipopolysaccharide-Induced Mouse Macrophages via Downregulation of the MAPK/NF-B Signaling Pathway. J. Chem..

[69] Yoon N.Y., Lee S.H., Wijesekara I., Kim S.K. (2011). In vitro and intracellular antioxidant activities of brown alga *Eisenia bicyclis*. Fish. Aquat. Sci..

[70] Peng J., Yuan J.P., Wu C.F., Wang J.H. (2011). Fucoxanthin, a marine carotenoid present in brown seaweeds and diatoms: Metabolism and bioactivities relevant to human health. Mar. Drugs.

[71] Ibañez E., Kubátová A., Señoráns F.J., Cavero S., Reglero U., Hawthorne S.B. (2003). Subcritical water extraction of antioxidant compounds from rosemary plants. J. Agric. Food Chem..

[72] Ko M.J., Cheigh C.I., Chung M.S. (2014). Relationship analysis between flavonoids structure and subcritical water extraction (SWE). Food Chem..

[73] Belda M., Sanchez D., Bover E., Prieto B., Padrón C., Cejalvo D., Lloris J.M. (2016). Extraction of polyphenols in *Himanthalia elongata* and determination by high performance liquid chromatography with diode array detector prior to its potential use against oxidative stress. J. Chromatogr. B Anal. Technol. Biomed. Life Sci..

[74] Cox S., Hamilton Turley G., Rajauria G., Abu-Ghannam N., Jaiswal A.K. (2014). Antioxidant potential and antimicrobial efficacy of seaweed (*Himanthalia elongata*) extract in model food systems. J. Appl. Phycol..

[75] Fernández-Segovia I., Lerma-García M.J., Fuentes A., Barat J.M. (2018). Characterization of Spanish powdered seaweeds: Composition, antioxidant capacity and technological properties. Food Res. Int..

[76] Ciesarová Z., Kukurová K., Bednáriková A., Morales F.J. (2009). Effect of heat treatment and dough formulation on the formation of Maillard reaction products in fine bakery products—benefits and weak points. J. Food Nutr. Res..

[77] André R., Guedes L., Melo R., Ascensão L., Pacheco R., Vaz P.D., Serralheiro M.L. (2020). Effect of food preparations on in vitro bioactivities and chemical components of fucus vesiculosus. Foods.

[78] Choi J.S., Haulader S., Karki S., Jung H.J., Kim H.R., Jung H.A. (2015). Acetyl- and butyryl-cholinesterase inhibitory activities of the edible brown alga *Eisenia bicyclis*. Arch. Pharm. Res..

