# Supplementary material for: Evaluation of the Biological Potential of Himanthalia elongata (L.) S.F.Gray and Eisenia bicyclis (Kjellman) Setchell Subcritical Water Extracts
Source: Foods. 2022 Mar 3;11(5):746. doi: 10.3390/foods11050746 (PMC8909621; doi:10.3390/foods11050746)
Supplement: Supplementary file 1 [file foods-11-00746-s001.zip › foods-1573187-supplementary.pdf]

## Supplementary material

**Table S1.** Chemical names, molecular formulas, CAS numbers, and molecular weight of the studied pollutants.

| Pesticides and Pharmaceuticals | Analyte | Molecular Formula | CAS number | Molecular Weight (g/mol) |
|---|---|---|---|---|
| **Organochlorine pesticides** | α-,β-, γ-, δ-hexachlorocyclohexanes (HCH) | $C_6H_6Cl_6$ | 319-84-6<br>319-85-7<br>319-86-8 | 290.8 |
| | Hexachlorobenzene (HCB) | $C_6Cl_6$ | 118-74-1 | 284.8 |
| | 1-Chloro-2-[2,2,2-trichloro-1-(4-chlorophenyl)ethyl]benzene (o,*p´*-DDT) | $C_{14}H_9Cl_5$ | 50-29-3 | 354.5 |
| | [2,2bis(p-chlorophenyl)-1,1-dichloroethylene] (*p,p´*-DDE) | $C_{14}H_8Cl_4$ | 72-55-9 | 318.0 |
| | Tetrachlorodiphenylethane (*p,p´*-DDD) | $C_{14}H_{10}Cl_4$ | 72-54-8 | 320.0 |
| | Aldrin | $C_{12}H_8Cl_6$ | 309-00-2 | 364.9 |
| | Dieldrin | $C_{12}H_8Cl_6O$ | 60-57-1 | 380.9 |
| | Endrin | $C_{12}H_8Cl_6O$ | 72-20-8 | 380.9 |
| | α, β-Endosulfan | $C_9H_6Cl_6O_3S$ | 959-98-8<br>33213-65-9 | 406.9 |
| | Methoxychlor | $C_{16}H_{15}Cl_3O2$ | 72-43-5 | 345.6 |
| **Organophosphorus pesticides** | Dimethoate | $C_5H_{12}NO_3PS_2$ | 60-51-5 | 229.3 |
| | Diazinon | $C_{12}H_{21}N_2O_3PS$ | 333-41-5 | 304.35 |
| | Chlorpyrifos-methyl | $C_7H_7Cl_3NO_3PS$ | 5598-13-0 | 322.5 |
| | Parathion-methyl | $C_8H_{10}NO_5PS$ | 298-00-0 | 263.21 |
| | Malathion | $C_{10}H_{19}O_6PS_2$ | 121-75-5 | 330.4 |
| | Chlorpyrifos | $C_9H_{11}Cl_3NO_3PS$ | 2921-88-2 | 350.6 |
| | Chlorfenvinphos | $C_{12}H_{14}Cl_3O_4P$ | 470-90-6 | 359.6 |
| **Psychiatric drugs and its metabolites** | Carbamazepine | $C_{15}H_{12}N_2O$ | 298-46-4 | 236.27 |
| | Citalopram | $C_{20}H_{21}FN_2O$ | 59729-33-8 | 324.40 |
| | Diazepam | $C_{16}H_{13}ClN_2O$ | 439-14-5 | 284.74 |
| | Fluoxetine hydrochloride | $C_{17}H_{19}ClF_3NO$ | 56296-78-7 | 345.79 |
| | Paroxetine hydrochloride | $C_{19}H_{21}ClFNO_3$ | 78246-49-8 | 365.83 |
| | Sertraline hydrochloride | $C_{17}H_{18}Cl_3N$ | 79559-97-0 | 342.69 |
| | Trazodone hydrochloride | $C_{19}H_{23}Cl_2N_5O$ | 19666-36-5 | 408.33 |
| | Venlafaxine hydrochloride | $C_{17}H_{28}ClNO_2$ | 99300-78-4 | 313.87 |

| | | | | |
|---|---|---|---|---|
| | Citalopram N-oxide hydrochloride (Citalopram metabolite) | $C_{20}H_{22}ClFN_2O_2$ | 62498-71-9 | 376.86 |
| | Citalopram propionic acid (Citalopram metabolite) | $C_{18}H_{14}FNO_3$ | **Not Available** | 311.31 |
| | Demethylcitalopram hydrochloride (Citalopram metabolite) | $C_{19}H_{20}ClFN_2O$ | 97743-99-2 | 346.83 |
| | Didemethylcitalopram hydrochloride (Citalopram metabolite) | $C_{18}H_{18}ClFN_2O$ | 1189694-81-2 | 332.80 |
| | *O*-Desmethylvenlafaxine (Venlafaxine metabolite) | $C_{16}H_{25}NO_2$ | 93413-62-8 | 263.38 |
| | 10,11-Epoxy carbamazepine (Carbamazepine metabolite) | $C_{15}H_{12}N_2O_2$ | 36507-30-9 | 252.27 |
| | Norfluoxetine hydrochloride (Fluoxetine metabolite) | $C_{16}H_{17}ClF_3NO$ | 57226-68-3 | 331.76 |
| | Norsertraline hydrochloride (Sertraline metabolite) | $C_{16}H_{16}Cl_3N$ | 675126-08-6 | 328.66 |
| **Pharmaceuticals used in Alzheimer's and Parkinson's diseases** | Amantadine hydrochloride | $C_{10}H_{17}N$ HCl | 665-66-7 | 187.71 |
| | Apomorphine hydrochloride | $C_{17}H_{17}NO_2$ HCl 1/2$H_2O$ | 41372-20-7 | 312.79 |
| | Benserazide hydrochloride | $C_{10}H_{15}N_3O_5$ HCl | 14919-77-8 | 293.70 |
| | Carbidopa | $C_{10}H_{14}N_2O_4$ $H_2O$ | 38821-49-7 | 244.24 |
| | Entacapone | $C_{14}H_{15}N_3O_5$ | 130929-57-6 | 305.29 |
| | R(-)-Deprenyl hydrochloride (Selegiline hydrochloride) | $C_{13}H_{17}N$ HCl | 14611-52-0 | 223.74 |
| | Donepezil hydrochloride | $C_{24}H_{29}NO_3$ HCl | 120011-70-3 | 415.95 |
| | Galanthamine hydrochloride | $C_{17}H_{21}NO_3$ HBr | 1953-04-4 | 368.27 |
| | Pramipexole dihydrochloride monohydrate | $C_{10}H_{17}N_3S$ 2HCl $H_2O$ | 191217-81-9 | 302.26 |
| | Safinamide mesylate salt | $C_{17}H_{19}FN_2O_2$ x$CH_4O_3S$ | 202825-46-5 | 302.34 |
| | Rasagiline mesylate | $C_{12}H_{13}N$ $CH_4O_3S$ | 161735-79-1 | 267.34 |
| | Rivastigmine hydrogen tartrate | $C_{14}H_{22}N_2O_2$ $C_4H_6O_6$ | 129101-54-8 | 400.42 |
| | Ropinirole hydrochloride | $C_{16}H_{24}N_2O$ HCl | 91374-20-8 | 296.84 |
| | Rotigotine hydrochloride | $C_{19}H_{25}NOS \cdot HCl$ | 125572-93-2 | 351.93 |
